# Dynamic prioritization of COVID-19 vaccines when social distancing is limited for essential workers

**DOI:** 10.1073/pnas.2025786118

**Published:** 2021-04-02

**Authors:** Jack H. Buckner, Gerardo Chowell, Michael R. Springborn

**Affiliations:** ^a^Graduate Group in Ecology, University of California, Davis, CA 95616;; ^b^Department of Population Health Sciences, School of Public Health, Georgia State University, Atlanta, GA 30303;; ^c^Department of Environmental Science and Policy, University of California, Davis, CA 95616

**Keywords:** COVID-19, vaccine prioritization, essential workers

## Abstract

COVID-19 vaccines have been authorized in multiple countries, and more are under rapid development. Careful design of a vaccine prioritization strategy across sociodemographic groups is a crucial public policy challenge given that 1) vaccine supply will be constrained for the first several months of the vaccination campaign, 2) there are stark differences in transmission and severity of impacts from severe acute respiratory syndrome coronavirus 2 (SARS-CoV-2) across groups, and 3) SARS-CoV-2 differs markedly from previous pandemic viruses. We assess the optimal allocation of a limited vaccine supply in the United States across groups differentiated by age and essential worker status, which constrains opportunities for social distancing. We model transmission dynamics using a compartmental model parameterized to capture current understanding of the epidemiological characteristics of COVID-19, including key sources of group heterogeneity (susceptibility, severity, and contact rates). We investigate three alternative policy objectives (minimizing infections, years of life lost, or deaths) and model a dynamic strategy that evolves with the population epidemiological status. We find that this temporal flexibility contributes substantially to public health goals. Older essential workers are typically targeted first. However, depending on the objective, younger essential workers are prioritized to control spread or seniors to directly control mortality. When the objective is minimizing deaths, relative to an untargeted approach, prioritization averts deaths on a range between 20,000 (when nonpharmaceutical interventions are strong) and 300,000 (when these interventions are weak). We illustrate how optimal prioritization is sensitive to several factors, most notably, vaccine effectiveness and supply, rate of transmission, and the magnitude of initial infections.

## Significance

Vaccines are a key intervention to reduce the burden of the COVID-19 pandemic. However, vaccine supply and administration capacity will initially be limited. Due to these constraints, it is critical to understand how vaccine deployment can be targeted to minimize the overall burden of disease. In this paper, we solve for optimal dynamic strategies to allocate a limited supply of vaccines over a population differentiated by age and essential worker status that minimizes the number of total deaths, years of life lost, or infections. We find that older essential workers are typically targeted first. However, depending on the objective and alternative model scenarios considered, younger essential workers may be prioritized to control spread or seniors to directly control mortality.

As the novel coronavirus (severe acute respiratory syndrome coronavirus 2 [SARS-CoV-2]) continues to inflict substantial morbidity and mortality around the world despite intervention efforts, public health experts see vaccines as essential to dramatically reduce the mortality burden and possibly halt local transmission (1). COVID-19 has resulted in over 2.3 million confirmed deaths globally (2) as of early February 2021. Fortunately, multiple promising vaccines are under rapid development, with the final weeks of 2020 seeing the first authorization and shipping of doses (3). However, vaccine availability will be highly constrained for at least several months (4). This scarcity, combined with stark differences in the spread and impact of SARS-CoV-2 across demographic groups, means that vaccine prioritization poses a key public health challenge. National and international public health organizations have mobilized to assemble guidance, including the World Health Organization (WHO), the National Academy of Medicine, and the Advisory Committee on Immunization Practices (ACIP) of the US Centers for Disease Control and Prevention (CDC) (5).

An effective public health policy for pandemic vaccine allocation requires an understanding of how risk of infection and severe disease varies across sociodemographic groups and how a given vaccine policy will impact the continued spread of infections within the population. Accounting for these two processes is critical when the population with the greatest risk of infection differs from those with the greatest risk of severe disease, as is the case for COVID-19, because an effective policy will need to balance direct protection of the most vulnerable against limiting secondary infections and rapidly achieving herd immunity (6). These key components can be integrated into a mathematical and statistical modeling framework of the transmission dynamics of the novel pathogen. Such an analytic framework can then be utilized to investigate the optimal vaccine allocation strategies to achieve a defined public health objective while taking into account the value of vaccines for mitigating health outcomes at the individual and population level.

Previous experience with vaccine development midpandemic offers limited insights for SARS-CoV-2 prioritization. SARS and Zika vaccine development was incomplete when those outbreaks ended (7). In 2009, as the novel A/H1N1 influenza virus continued to spread across the United States, researchers investigated optimal vaccination strategies using an age-structured dynamical model. They found that school-aged children and their parents should be prioritized, a strategy that would indirectly protect individuals at higher risk of severe health outcomes (8). Sharp differences in the epidemiology of human influenza and COVID-19 indicate that vaccination strategies against the ongoing pandemic should not simply mirror vaccination policies against influenza. For example, COVID-19 is associated with lower susceptibility to infection among children and adolescents (9, 10) and has a substantially higher infection fatality rate overall that also increases markedly with age (11). Toner et al. (ref. (5), p. 24) provide a detailed overview of the 2018 pandemic influenza vaccination plan and conclude that “the priority scheme envisioned … does not comport with the realities of the COVID-19 pandemic and new guidance is needed.” Fitzpatrick and Galvani (12) concur, detailing how the unique “epidemiological, clinical, behavioral, and vaccine-related relationships” of SARS-CoV-2 motivate the need for “pathogen-specific transmission modeling.”

**Fig. 1.**
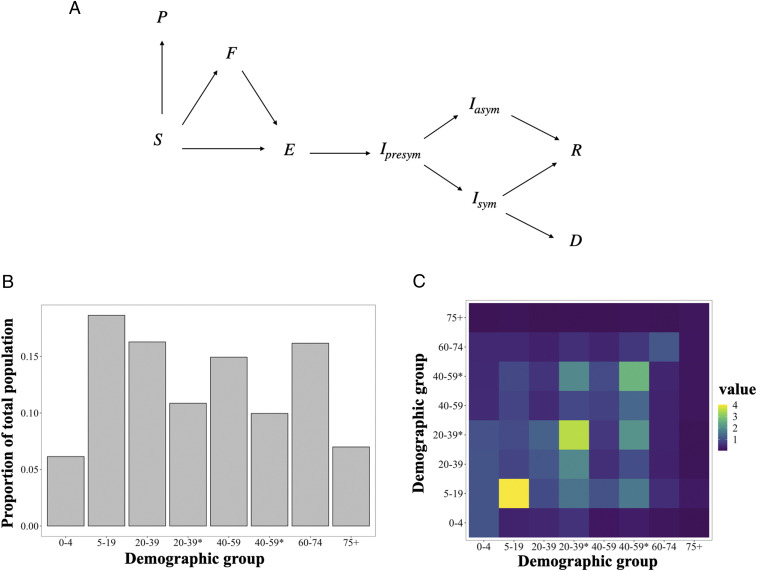
Schematic of the modeled movement of individuals between epidemiological states defined in *Methods* (*A*), the portion of individuals from the US population in each demographic group determined by essential worker status (*) and age (*B*), and the contact rates between demographic groups, given by average daily number of contacts a group on the horizontal axis makes with a group on the vertical axis (*C*).

We develop and apply a mathematical model to assess the optimal allocation of limited COVID-19 vaccine supply in the United States across sociodemographic groups differentiated by age and essential worker status (see *Methods*). The transmission dynamics are modeled using a compartmental model tracking eight demographic groups through the nine disease states as shown in Fig. 1. The parameters are calibrated to capture our current understanding of the epidemiology of COVID-19, and our analysis is designed to capture two key features of COVID-19 prioritization: essential workers and the gradual availability of vaccines over time. A large number of workers are constrained in their ability to work from home (essential workers), exposing them to a higher level of risk of infection, and increasing the chance they transmit the disease if infected. Policies that account for the greater risk essential workers are exposed to may be more just and highlight a group of individuals “who have been overlooked in previous allocation schemes” (5). Furthermore, these policies may be more effective at mitigating morbidity and mortality, as they can account for a key factor driving transmission of the disease.

To account for the gradual rollout of vaccines, we employ stochastic nonlinear programming techniques to solve for vaccine prioritization policies that distribute vaccine to susceptible individuals and change on a monthly time step responding to changes in the epidemiological status of the population (shares of the population in different disease states). These dynamic policies account for a key feature of the policy-making process, since the supply of vaccine is likely to be constrained, with available doses administered as they become available over a period of several months.

The transmission of COVID-19 is a complex process contingent on the characteristics of the disease and ever-changing social behavior. Furthermore, many of the key dynamics can change depending on the spatial scale considered, with differences in the transmission process within and between communities. We seek to summarize the features of the complex and evolving processes that are most relevant to the spread of the disease within and between sociodemographic groups. To do so, we model COVID-19 transmission with the social contact hypothesis (13) and describe the contact patterns between demographic groups using contact matrices estimated for the United States from Prem et al. (14) scaled by the location where the contacts were made (home, school, work, and other) to reflect the impacts of social distancing. Although these assumptions present a stylized version of contacts during the pandemic, they allow us to capture many key features of social contacts, such as the concentration of contacts within age groups, parent–child relationships, and receiver–caregiver relationships (15).

Existing published studies of COVID-19 vaccination prioritization analyses include Matrajt et al. (16) and Bubar et al. (17).^†^ Both consider the optimal allocation of vaccines across five or more age groups within a country. Their approaches feature rich exploration of policy sensitivity to vaccine effectiveness and availability. Matrajt et al. are particularly detailed in this respect, while Bubar et al. uniquely consider differences in demographics and contact rates across multiple countries, and Hogan et al. (19) also consider allocation between countries. Our analysis is differentiated by a deeper approach to the behavioral, demographic, and decision models by addressing social distancing, essential worker groups, and allocation policies that can change over the course of the vaccination campaign.

General ethical guiding frameworks for vaccine prioritization decision-making have appeared earlier in the literature. Toner et al. (5) emphasize promoting three ethical values: the common good; fairness and equity; and legitimacy, trust, and communal contributions to decision-making. Emanuel et al. (4) promote four ethical values: maximizing benefits, treating equally, instrumental value, and priority to the worst off. Our analytic focus on minimizing new infections, years of life lost (YLL), or deaths emerges from promoting “the common good” or “maximizing benefits.” Our focus on essential worker groups illustrates how ethical values (e.g., prioritizing essential workers due to the fairness of protecting those placing themselves at risk) may overlap with the common good (e.g., prioritizing essential workers to best reduce mortality and transmission). Issues of fairness and equity and protecting the worst off are not directly analyzed here but remain critical considerations.

For the sake of simplicity, we do not address, in detail, the potential set of complex and differential feedback processes between health status and opening of schools, workplaces, and other institutions. While we limit policy objectives to a concise metric of health outcomes (minimizing expected cases, YLL, or deaths), we acknowledge that other values of returning to school, work, and social life are important. Finally, we do not address additional vaccine complications, such as temporary effectiveness, potential side effects, or any failure to take a second dose of the vaccine if necessary.

Although much is known about the epidemiology of COVID-19, uncertainty remains a key limitation to modeling the disease. Therefore, we consider a wide range of plausible scenarios and focus on the general features of the solutions, the commonalities between the alternative scenarios, and identification of model parameters that drive systematic differences in optimal vaccine allocations.

Given these assumptions, we find that optimal allocation strategies are responsive to both the initial and evolving epidemiological landscape of the disease. When focusing on mortality (YLL or deaths), vaccination of older essential workers and ages 60+ y was almost always a top priority (i.e., targeted in the first 30% of the population vaccinated). Alternatively, when infections are minimized, essential workers are prioritized, followed by school-age children, across a range of likely scenarios. We find that prioritization can substantially improve public health outcomes—31 to 40% in the base scenario, relative to untargeted vaccination. Two components unique to our model are important contributors to this improvement. First, policies that differentiate and target essential workers in addition to age substantially outperform those utilizing age alone. Furthermore, essential worker differentiation reduces trade-offs between objectives (e.g., deterioration of YLL and infection metrics when focused on minimizing deaths). Second, extending from a static allocation (without phases) to allowing changes in prioritization over time provides substantial gains. Finally, while optimal prioritization is quite insensitive to model specification when minimizing infections, we find some sensitivity when focused on minimizing deaths or YLL. This sensitivity indicates benefits to adjusting the targeting strategy at the local level to match epidemiological conditions.

## Results

**Fig. 2.**
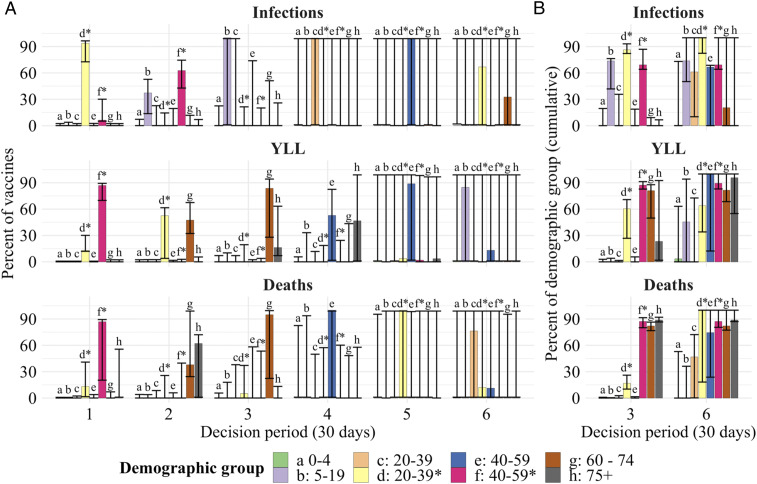
The optimal allocation of vaccines (vertical axes) between demographic groups for each decision period (horizontal axis) under the base scenario (*A*). The rows represent each objective, to minimize deaths (*Top*), minimize YLL (*Middle*), and minimize infections (*Bottom*). The bars for the six decision periods show the percentage of vaccines allocated to a specific group (indicated by a letter, color, and asterisks indicating essential worker groups) in that period. *B* shows cumulative measures at the end of months 3 and 6, respectively, for the percent of each group that has been vaccinated. The whiskers on each bar represent the sensitivity of the optimal solution to small deviations in the outcome, specifically, the range of allocations resulting in outcomes within 0.5% of the optimal solution.

To illustrate the qualitative nature of optimal dynamic prioritization, we first present results from a single “base” scenario, representing a plausible set of parameters (detailed in *SI Appendix*, Table S1). These results are then compared to a set of alternative model scenarios as described in Table 1. While we begin with base scenario results, we emphasize the sensitivity analysis under alternative scenarios that follows, since information about some input parameters—for example, expected vaccination supply—continues to change with time. In Fig. 2, the base model allocation decisions are shown for each monthly decision period (in percent of vaccine supply) and then cumulatively (in percent of group vaccinated) at 3 and 6 mo, respectively. Broadly, we find that the optimal policy is very dynamic: Specific groups are targeted each period, and these targets shift over time. Furthermore, targeting is very narrow initially, but then becomes less so as a larger fraction of the population has been covered.

**Table 1.** Descriptions of alternative scenarios relative to the base model (see *SI Appendix*, section A for specific levels)

Scenario	Change from base scenario parameters	Source
Base scenario	None (Base parameter values are provided	
	in *SI Appendix*, section A)	
High initial infections	Increased number of initial symptomatic	Assumed: pandemic state will
	infections (300% increase)	vary between localities when
		vaccine first available to the
		general public
Strong NPI	NSD NPI are strong, resulting in	Consistent with R<1
	a declining infection rate	
Weak NPI	NSD NPI are weak, resulting in a sharply	Consistent with R≫1
	increasing burden of infection	
Weak vaccine	Lower vaccine effectiveness (success rate) for	Minimum value required by
	all age groups relative to the base scenario	FDA guidelines
Weak vaccine 60+	Lower vaccine effectiveness for	Informed by influenza vaccine
	ages 60+ y	effectiveness
Even susceptibility	All ages are equally susceptible to infection;	Assumed: tests sensitivity
	increase in susceptibility for ages <20y	to age-dependent susceptibility
	relative to Base	described by refs. 9 and 20
Low supply	Sufficient supply for 5% of the population	Assumed: vaccine supply is
	monthly (50% of supply relative to base	uncertain and known to impact
	scenario; prioritization changes every 10% of the	optimal allocations (21)
	population vaccinated, such that decision	
	period is 2 mo)	
Ramp up	Vaccine supply is 5% per month for	Informed by comments from
	the first 2 mo and 10% per month thereafter	the scientific head of the US
	(first decision period is 2 mo, so increments	vaccine development
	of 10% of the population are vaccinated each	program (22)
	decision period)	
Open schools	Rate of social contact in schools increased	Assumed: tests sensitivity of
	from 30% in base model to 70%	optimal allocations to school
		closure intensity
High contacts	Increased number of contacts outside	Assumed: tests sensitivity to
	the home, school and workplace (50% increase	relaxed distancing
	relative to base)	

The whiskers on bars in Fig. 2 show the range of alternative allocations that still produce an outcome that is within 0.5% of the optimum. These indicate that the optimized outcome is relatively sensitive to substitutions between groups for the first 3 mo, as indicated by narrow whiskers around the cumulative allocations. There is, however, some limited ability to substitute vaccines between the two essential worker groups in the first 2 mo when minimizing YLL or deaths. As the size of the susceptible population declines due to vaccination and infections, the optimized outcomes become less sensitive to substitutions (longer whiskers), with shifts between nearly all groups possible without substantial sacrifice. This suggests that targeting strategies can become less strict over time as the most vulnerable populations are protected. Comparing individual periods (Fig. 2*A*) and cumulative measures (Fig. 2*B*) shows that whiskers represent a combination of substitution between groups as well as between periods for the same group.

Across objectives, there are substantial differences in which groups are targeted early on. When minimizing deaths, targeting progresses from essential workers (20 y to 39 y*, 40 y to 59 y*), to the oldest (75+ y), and then younger seniors (60 y to 74 y) (Fig. 2). These groups are a mix of those at high risk of mortality (older groups) and high risk of contraction and spread (essential workers). When minimizing YLL, younger seniors are targeted earlier (given their longer average years of life remaining).^‡^ Finally, when minimizing infections, we find that younger essential workers take top priority, followed by older essential workers and school-age children (5 y to 19 y), since these groups have higher contacts and thus higher risk of contraction and spread.

**Fig. 3.**
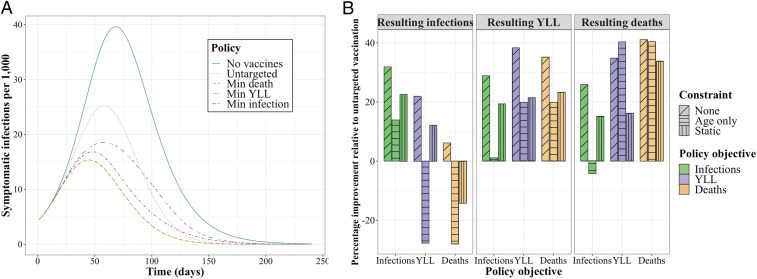
The number of infections per 1,000 individuals over time under reference policies (no vaccines; untargeted vaccine allocation) and optimized policies minimizing (min) a given metric (*A*) and the performance of each optimized policy relative to an untargeted allocation policy (*B*) for the base scenario. The bars are boxed by each resulting metric, colored by the objective driving each policy, and textured to reflect any constraint considered (e.g., age-only or static policies).

In Fig. 3*A*, we show the dynamic path of infections, starting from the period in which vaccines become available, under various policies. As expected, infections are highest given no vaccines. Results for allocating vaccines in a manner proportional to each group’s size shows the substantial value of even “untargeted” vaccines. As expected, the policy for minimizing infections leads to the lowest level of infections.

In Fig. 3*B*, we show the performance of various policies for resulting outcome metrics (infections, YLL, and deaths) in terms of the percentage improvement relative to an untargeted vaccine allocation. We consider the optimal policies presented in Fig. 2 where the objective is minimizing infections (green), YLL (purple), or deaths (orange) with no constraints (“none”). We also consider two constrained alternatives: an “age-only” dynamic policy that does not differentiate by essential worker status, and a “static” policy where the fractional allocation across groups does not change over decision periods.^§^ We find that the unconstrained policy—that is dynamic and differentiated by essential workers—outperforms the untargeted approach by approximately 31 to 40% depending on the objective. Relative to the unconstrained policy, the age-only and static policies perform substantially worse for infections and YLL, although not for deaths. However, even while the age-only and static policies do not substantially impede performance in minimizing deaths, these constrained approaches still suffer substantial performance loss (9 to 18 percentage points) in the other two outcomes not optimized (YLL and infections) but clearly still of interest.^¶^ In other words, accounting for both essential workers and a dynamic prioritization strategy provides substantial improvements in the metric being optimized and/or the other two metrics of interest.

In general, we find that, no matter the policy objective pursued in targeted vaccine allocation, some improvement is made on all three metrics. However, there are trade-offs in what can be achieved between the objectives. For example, policies that minimize infections result in substantially more deaths than a policy that minimizes deaths. We also find that differentiating essential workers substantially reduces these trade-offs between objectives relative to age-only or static policies.

### Sensitivity of Vaccine Prioritization.

To assess how robust our base scenario findings are to key uncertainties in the model, we conduct three different sensitivity analyses. First, we consider a set of 10 alternative plausible scenarios involving a broad set of model inputs; then, we focus on a narrower set of four parameters, each explored in richer gradient detail; finally, we examine a few fundamental changes to model structures.

#### A broad set of alternative scenarios.

We solved for the optimal vaccine allocation across a range of 10 alternative scenarios selected to assess sensitivity to key assumptions of the base model. Differences between these scenarios and the base case are detailed in *SI Appendix*, Table S1. Relative to the base model, in these alternative scenarios, we consider higher initial infections, stronger or weaker nonsocial distancing (NSD) nonpharmaceutical interventions (NPI) like mask wearing, weaker vaccine effectiveness overall or for seniors (60+ y), lower vaccine supply or supply that starts low and ramps up, more open schools, or higher contact rates overall.

**Fig. 4.**
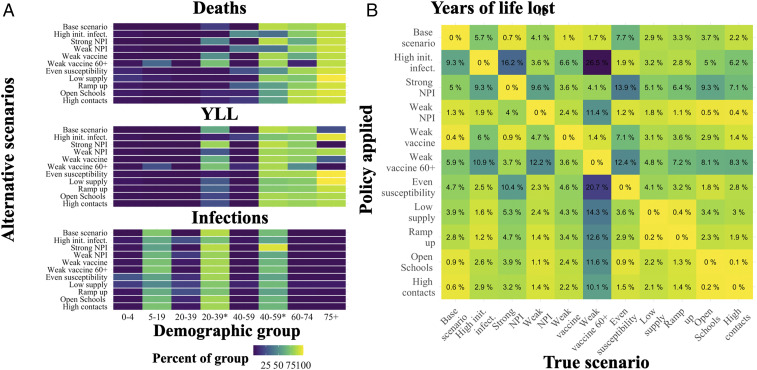
The cumulative percent of each demographic group (horizontal axis) vaccinated after the first 30% of the population is vaccinated under the alternative scenarios (vertical axis) and each objective to be minimized including deaths (*Top*), YLL (*Middle*), and infections (*Bottom*) (*A*); init. infect., initial infections. The percentage of additional YLL in excess of the optimum when applying a policy for a given alternative scenario (row) when a particular scenario is the “truth” (column) (*B*). *, indicating essential worker groups.

To compare and contrast optimal early vaccination allocation for each scenario and objective, in Fig. 4*A*, we show the percentage of each group vaccinated after 30% of the overall population is covered (typically in 3 mo, except for alternative supply scenarios). We find that high priority groups—by percent of group vaccinated—are typically but not always robust to the alternative scenarios. For example, when deaths are considered (Fig. 4 *A*, *Top*), we see substitution between younger essential workers (29 y to 39 y*) and ages 60 y to 74 y, and, when YLL are considered, there is substitution between younger essential workers and ages 75+ y. To illustrate differences in the relative order of these high-priority groups, in *SI Appendix*, Fig. S5, we show optimal prioritization of vaccination in the very first decision period across objectives and scenarios. We find that, when YLL are considered, essential workers ages 40 y to 59 y are the highest-priority group in all scenarios. However, when deaths are considered, ages 75+ y are the highest-priority group under several alternative scenarios.

For insight into the cost of error in specifying the correct scenario, we assessed the performance of the policy identified for each of the 11 alternative scenarios, depending on which of these 11 is the “true” scenario. In Fig. 4*B*, we show these results for the YLL objective. For example, the first column shows the performance loss (in percentage of additional YLL above the optimum) when the true scenario is the base model but the decision maker applies a policy matched to any of the alternative scenarios (rows). By construction, when the policy applied matches the true scenario, the performance loss is zero. When YLL is the focus and the base specification is the “true” scenario, the greatest performance loss (9%) comes from mistakenly applying the high initial infections policy.

We find that performance costs, in percentage terms, from applying the wrong policy from this set are typically modest (low single digits), albeit with notable exceptions. For example, when the “truth” is that we have a weak vaccine for ages 60+ y, several policies applied perform very poorly relative to the true optimal policy, since they substitute vaccine away from younger essential workers to ages 75+ y. A few of the policies were generally less robust across various true models, specifically, those for high initial infections, strong NPI, and weak vaccine 60+. The base scenario policy performed reasonably well across true alternative models, with the largest loss arising (7%) when children are not less susceptible (even susceptibility).

Equivalent versions of Fig. 4*B* for minimizing deaths or infections are provided in *SI Appendix*, section C. When the focus is minimizing deaths, the pattern of performance between scenarios is very consistent with YLL in Fig. 4. However, the scope for performance loss is larger overall—up from a maximum of 26% for YLL to 46% for deaths. When the focus is infections, the range of performance loss is much less intense, at 7%. For infections, this relatively robust performance arises because optimal policies are much more similar across scenarios when minimizing infections (compared to the other objectives). Given greater scenario-driven heterogeneity in policies for minimizing YLL or deaths, there is greater opportunity for performance loss from specification error.

#### A gradient over four key parameters.

**Fig. 5.**
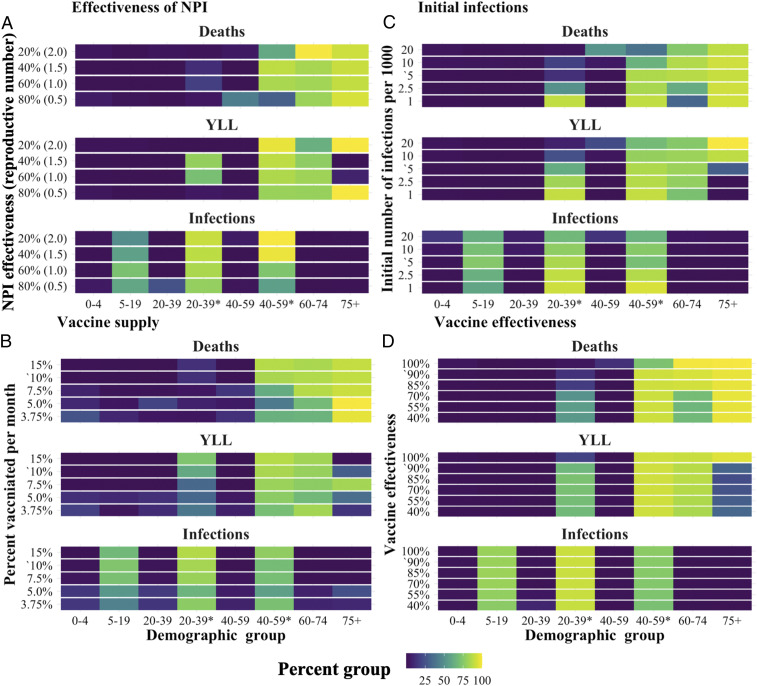
The total percent of each demographic group vaccinated after 3 mo under the optimal dynamic policy. Each panel shows the effect of varying a key parameter relative to the base model: (*A*) effectiveness of NPI, which determines the initial reproductive number (when the vaccine first becomes available); (*B*) monthly rate of vaccine supply; (*C*) initial infections; and (*D*) vaccine effectiveness. Base scenario parameter values are indicated with an apostrophe (‘). *, indicating essential worker groups.

For further sensitivity analysis, as shown in Fig. 5, we assessed how optimal vaccine allocation policy changed along a gradient for four key model inputs: NSD NPI effectiveness (e.g., mask wearing) which determines the initial reproductive number (when the vaccine first becomes available), initial infections, monthly rate of vaccine supply, and vaccine effectiveness.

Echoing sensitivity results reported above, variation in these parameters had little effect on the optimal policy for minimizing infections. But we found systematic differences in the policies for minimizing YLL and deaths. Essential workers, ages 60 y to 74 y and ages 75+ y, remained the highest-priority groups across the full range of parameters tested, but there was substitution between younger essential workers (29 y to 39 y*) and the older age groups.

In most instances, the percent of vaccines responded in relatively monotonic fashion as parameters varied. For example, consider the objective of minimizing deaths. As depicted in Fig. 5, prioritization of essential workers fell and 60+ y or 75+ y increased as 1) initial infections grow, 2) vaccine supply decreases, or 3) vaccine effectiveness increases. In a few instances, the percent of vaccine allocated to a given group responded nonmonotonically to variation in the parameter. For example, for effectiveness of NPI in Fig. 5*A*, the allocation skewed toward 75+ y and away from essential workers when the parameter was very high and very low.

These results indicate that, when focusing on deaths or YLL, if transmission cannot be reduced quickly by the vaccine—due to limited supply, high reproductive numbers, or large initial number of infections—typically, this initial supply is most efficiently used to directly protect individuals with the greatest risk of death if infected. This pattern differs for vaccine effectiveness: We find that, as the effectiveness of the vaccine decreased, supply is substituted away from the older (higher risk) age groups to essential workers. This difference is consistent with the fact that, as vaccines become less effective for a given individual, protecting vulnerable individuals is better achieved by reducing population-level transmission.

#### Changes to model structures.

As a final sensitivity analysis, we examined robustness of the results to three alternative model structures: 1) clustered essential workers, where essential workers only contact other essential workers in the workplace; 2) concentrated essential workers, where, relative to the baseline scenario, the portion of the working age population deemed “essential” is half (20%), and they have approximately double the contact rate; and 3) leaky vaccine, where, rather than working perfectly for 90% of individuals, vaccinated individuals have reduced susceptibility to infection, infectiousness, and risk of death if infected. A more detailed discussion of these models is included in *SI Appendix*, section D.

We found that the qualitative nature of the solutions remained constant across each of these alternative models, with some minor differences. Treating the essential worker group as a cluster increased the proportion of vaccine allocated to ages 60+ y when deaths and YLL were considered. This shows that, when essential worker contacts are clustered within group, this reduces the indirect protection that vaccinating these individuals provided to others. Conversely, concentrating the essential worker group (to a more select group with higher contact rates) increased the fraction of these individuals vaccinated. This shows that select essential workers with especially high contact rates (e.g., medical professionals and essential retail workers) are particularly strong candidates for early vaccination.

## Discussion

Key insights and results from our analysis are summarized in Box 1. Together, these lessons show the strong implications of considering dynamic solutions, social distancing, and essential workers (given their limitations in social distancing) for vaccine prioritization.

Box 1 Key insights and results

1) **Benefits:** Prioritization can reduce a particular undesirable outcome (deaths, YLL, or infections), by 32 to 40% in the base scenario (or 17 to 44%, depending on the alternative scenario).
2) **Objectives:** Moving from minimizing infections to YLL to deaths boosts each of the following: benefits from vaccination targeting, prioritization differences between scenarios, and (therefore) the sensitivity of optimal prioritization to scenario.
3) **Dynamic prioritization:** Dynamic prioritization 1) is responsive to the initial and evolving disease status and 2) generates substantial improvement in outcomes relative to a static prioritization, indicating that a phased approach to vaccine distribution is well justified. However, diminishing marginal returns to additional vaccination within a group drives a shift to other groups before 100% vaccination of the first group is achieved.
4) **Widening prioritization:** As vaccination rates rise, precise prioritization becomes less critical, and targeting widens to a larger set of groups.
5) **Trade-offs:** Policies that target one objective forgo opportunities to reduce alternative metrics. For example, policies that minimize deaths do not reduce infections nearly to the same degree as policies that minimize infections. These trade-offs are typically stronger when policies do not allow for targeting based on essential worker status.
6) **Essential workers:** Relative to an age-only model, policies that allow targeting of essential workers provide the greatest improvements when minimizing infections and YLL are the focus. In the base scenario, essential workers are a high priority group under all three objectives (i.e., they are among the first 30% of the population to receive vaccines). However, their priority relative to ages 60+ y is affected by key model parameters (see **Sensitivity** next).
7) **Sensitivity:** The high-priority groups remain consistent across the range of parameters considered. However, when minimizing deaths or YLL, the fraction of vaccine allocated to essential workers and ages 60+ y depends on the number of infections and reproductive number when the vaccine became available, the supply of vaccines, and vaccine effectiveness. In effect, when the vaccine has a limited ability to quickly reduce the transmission of the virus, optimal policies more heavily prioritize older individuals.

Our analysis of COVID-19 vaccine prioritization uniquely accounts for two critical needs: 1) dynamic prioritization given gradual rollout of vaccine during an active pandemic, and 2) attending to substantial heterogeneities in work contacts among the adult population due to the ability of many to work from home. These two novel features demonstrably change optimal vaccine prioritization. Given gradual vaccine deployment, static policies are out-performed by dynamic policies, which narrowly target a small number of demographic groups and (after substantial coverage of them) switch to lower-priority groups. Static policies identify a set of high-priority groups but not how to order them when phased deployment is necessary. More strikingly, targeting essential workers (or other adults with a large number of work contacts) reduces not just the adverse outcome of focus but also trade-offs for remaining outcomes. For example, when minimizing deaths, allocation that differentiates essential workers substantially lessens the degree to which infections and YLL climb from the minimum achieved when each is optimized on its own.

Existing published analysis of optimal COVID-19 vaccination targeting includes Matrajt et al. (16) and Bubar et al. (17). Before comparing and contrasting results, some key modeling differences should be noted. These two analyses consider a wider range of vaccine availability than considered here. Our analysis uniquely incorporates NPI, including social distancing and NSD (e.g., mask wearing). Doing so allows us to account for differences between groups like essential workers constrained in distancing versus others who are much less so. All three preprints implement static optimization where vaccines are allocated and administered in a one-shot process. Our allocation is dynamic, responding to changing epidemiological conditions over a 6-mo period. Finally, all three model vaccines as “leaky,” that is, reducing the probability that a susceptible individual will be infected [and the probability of severe disease (19)]. Bubar et al. also consider an “all-or-nothing” vaccine that is 100% effective for a fraction of the population. In our base model, the vaccine is “all-or-nothing,” although we also consider a leaky vaccine, as discussed at the end of *Results*.

Matrajt et al. (16) found that optimal strategies to minimize deaths and YLL will either exclusively target groups with high infection fatality rates, maximizing the direct benefit of vaccines, or will target groups with high rates of infection, maximizing the indirect benefits of the vaccine. In contrast, our results indicate that optimal policies initially target groups with high risk of infection and then switch to targeting groups with high infection fatality. This difference most likely follows from our dynamic versus static allocation. The switching behavior we identify is consistent with past work on pandemic influenza vaccine prioritization, which suggests that, early in an outbreak when the infection rate is growing, targeting spread (maximizing indirect benefits) is more efficient, but, later, when the infection rate is leveling off or declining, maximizing direct protection is most efficient (21).

Bubar et al. (17) found that prioritizing adults older than 60 y of age is a robust strategy for minimizing deaths. In contrast, we find that working-age adults are a key priority group, particularly older essential workers. These differences may arise from either our allowance for social distancing and/or dynamic allocation. Our accounting for social distancing on COVID-19 transmission increases the modeled benefits of targeting essential workers, who are less able to substantially reduce their social contacts than individuals over 60 y old. Furthermore, as discussed above, the ability of dynamic policies to switch over time allows the allocation schemes we discuss to capture the benefits of using the initial vaccine supply to slow transmission without sacrificing direct protection of more-vulnerable individuals later on.

Two notable additional analyses of optimal COVID-19 vaccination targeting in preprint form include Wang et al. (23), who focus on the mortality costs of delay in vaccine rollout and the trade-off between prioritizing first versus second doses, as well as Hogan et al. (19), who examine ideal allocation both within and between countries.

National and international institutions have also begun to disseminate guidance. In particular, general guidelines for vaccine prioritization have been put forward by Strategic Advisory Group of Experts of the WHO (24) and the US CDC’s ACIP (25). For example, CDC recommendations prioritize: 1) health care personnel; 2) residents of long-term care facilities; 3) persons aged 75 y and over and frontline essential workers; 4) persons aged 65 y to 74 y, persons aged 16 y to 64 y with high-risk medical conditions, and essential workers; and 5) everyone aged 16 y and over remaining. Categories A and B are subgroups at a finer scale than considered here, although with clear logic supporting top priority. Notably, a clear priority is not set between persons aged 75 y and over and frontline essential workers. This is consistent with our findings, in that prioritization within this pair was sensitive to specific conditions, which will vary over location. Our recommendations differ in the distinction made here between younger and older essential workers, with priority on the latter motivated by increasing mortality from infection with age. The CDC guidelines also consider underlying health conditions, a salient distinction not considered here. WHO guidelines—written more broadly for a global audience—agree on the prioritization of frontline health care workers at high risk of infection, followed by older adults. However, subsequent priority focuses on various sociodemographic groups at high risk (e.g., those in poverty) and essential educational workers before turning to essential workers more broadly.

Although our model provides useful insight for the policy-making process, a number of caveats are in order. In reality, the risk of infection varies continuously across individuals, even between different “essential” occupations. While our model is unique in capturing differences between essential and nonessential workers, the representation of these differences is simplified by averaging the total number of contacts over a group with high work contacts (essential workers) and a group with lower rates of work contacts. This allows us to demonstrate the importance of this heterogeneity in the adult population relative to the standard age-only models, indicating that policy makers should strongly consider occupation-differentiated vaccine allocation strategies.

While we explored a large set of alternative scenarios, further extensions remain for future work. For example, if certain groups (e.g., children or seniors) experience significant vaccination side effects, prioritization might shift away from these groups (26). From a logistical perspective, vaccination will occur through various points of contact with the community (pharmacies, clinics, schools, etc.). Constraints imposed by the distribution network used will affect the relative costs of reaching various subgroups. While the longevity of immunity to COVID-19—either following natural infection or vaccination—is not yet well understood, emerging analysis suggests that, following infection, “durable immunity against secondary COVID-19 disease is a possibility for most individuals” in the sense that immune memory was present in approximately 95% of individuals studied 5 mo to 8 mo after symptom onset (27). How long lasting immune memory will be in the longer run is a key unknown. We assume immunity spans at least through the end of our 6-mo time horizon. However, if, instead, this durability is more limited and/or already waning for those infected early in 2020, we might expect the symptomatic infections curve (Fig. 3*A*) to stretch farther out and for ideal vaccination strategies to shift toward direct protection of older, vulnerable populations.

From a behavioral perspective, vaccine hesitancy may influence the ability to achieve vaccination priorities, especially as coverage of the population increases. In general, we find that it is not necessary or even ideal to vaccinate all of the susceptible individuals in a demographic group, at least given the level of 60% of the population vaccinated considered here. Thus, at least initially, some level of vaccine hesitancy may have limited material impact. However, hesitancy may play a more significant role in the longer run, especially if hesitancy rates are large and herd immunity proves difficult to achieve (e.g., if vaccine effectiveness is low, and/or NPI relaxation is aggressive). Vaccine hesitancy that is concentrated in a particular community or demographic group could also change the optimal prioritization strategy. Similarly, adjustments would be needed if groups differ in the duration of vaccine effectiveness or diligence in obtaining a second dose of the vaccine where (and when) necessary.

For simplicity, we limited policy objectives to a set of concise metrics of health outcomes (minimizing expected cases, YLL, or deaths). However, other health-related metrics such as protecting the most vulnerable and social values such as returning to school, work, and social life are important to consider. Our analysis reveals that optimal strategies for minimizing deaths and YLL are broadly aligned with the goal of protecting the most vulnerable. These solutions target essential workers who are the least able to participate in NPI such as social distancing and thus are the most at risk for infection, and individuals over the age of 60 y who have the highest risk of death if infected by the disease. Other social values such as returning to school will most likely change the allocation schemes to offset the risk created by relaxing social distancing. For example, if allowing children to return to school was a high priority, then allocation strategies might be tilted toward targeting school-age children and teachers. A detailed analysis of optimal vaccine allocation given the relaxation of social distancing to achieve particular social objectives is a key direction for future research.

## Methods

### Model.

To investigate the impact of vaccination strategies on the COVID-19 pandemic in the United States, we employed a structured compartmental transmission model similar to ref. 28. We incorporated the demographic structure of the population by tracking six age groups in the set J={0to4,5to19,20to39,40to59,60to74,75+}. We then extend this set to differentiate essential workers by splitting the two prime working age groups into two groups—nonessential workers (20 y to 39 y, 40 y to 59 y) and essential workers (20 y to 39 y*, 40 y to 59 y*)—yielding four groups of prime working age individuals and a total of eight demographic groups in $J=\left\{0\text{to}4,5\text{to}19,20\text{to}39,20\text{to}39^{*},40\text{to}59,40\text{to}59^{*},60\text{to}74,75+\right\}$. For each demographic group, we tracked nine epidemiological states: susceptible (S), protected by a vaccine (P), vaccinated but unprotected (F), exposed (E), presymptomatic ($I_{pre}$), symptomatic ($I_{sym}$), asymptomatic ($I_{asym}$), recovered (R), and deceased (D). In Fig. 1, we display the compartmental diagram and directions of transitions between epidemiological states.

We modeled the COVID-19 transmission dynamics using a system of coupled ordinary differential equations for each demographic group, indexed by i and j. The transmission rate was given by the product of the transmission probability (q), the age-specific susceptibility ($s_{i}$), strength of NPIs (θ), the relative infectiousness of each symptom type ($\tau_{m}$)—where m∈M≡{asym,pre,sym}—and the rate of contact ($r_{m,i,j}$) between infected individuals with symptom type m from group j and susceptible individuals from group i. The exogenously given population vaccination rate at time t is given by v(t), where units of time are days.^#^ In our base model, we assume that, for each individual, the vaccine either works or it does not (although we also consider vaccines that are partially effective for all vaccinated in our sensitivity analysis). Individuals in group i are vaccinated at a rate of $\mu_{i}v\left(t\right)$, and a fraction of the those ($\epsilon_{i}$) are protected, while a fraction remain susceptible and move to the failed vaccination category (F).^∥^ Once infected, individuals move from exposed to presymptomatic at rate $\gamma_{exp}^{-1}$. Presymptomatic individuals become symptomatic or asymptomatic at rates $\sigma_{asym}/\gamma_{pre}$ and $\left(1-\sigma_{asym}\right)/\gamma_{pre}$, respectively. Asymptomatic individuals recover at an uniform rate $\gamma_{asym}^{-1}$, and symptomatic individuals either recover or die at a rate of $\left(1-\delta_{a}\right)/\gamma_{sym}$ or $\delta_{a}/\gamma_{sym}$, respectively, where $\delta_{a}$ is the age-specific infection fatality rate. These assumptions yield the system of differential equations described in *SI Appendix*, section A, with parameter values also given in *SI Appendix*, section A.

### Contact Rates.

Contact rates indicating the level of direct interaction of individuals within and between groups drive the transmission dynamics in the model. We built the contact matrices used in this model from the contact matrices estimated for the United States in ref. 14. These estimates are given for age groups with 5-y age increments from 0 y to 80 y. These estimates were aggregated to provide estimates for the six-level age structure used in our model. We also extended these data to estimate the contact rates of essential workers. A detailed derivation of these contact rates can be found in *SI Appendix*, section A. In short, we assumed that essential workers have, on average, the same pattern of contacts as an average worker in the population in the absence of social distancing. We then scaled the contact rates for essential and nonessential workers to represent the effects of social distancing, and calculated the resulting mixing patterns assuming homogeneity between these groups.

We constructed contact matrices for each of four locations, x∈{home,work,school,other}, following ref. 14. The total contact rate for an asymptomatic individual before the onset of the pandemic is given by the sum of these location-specific matrices. However, it is clear that populations are exhibiting social distancing in response to the pandemic (29). We further expect symptomatic individuals to change their behavior in response to the illness. We account for these behavioral changes as described next.

### Social Distancing.

Expression of symptoms and social distancing policies are likely to change individuals’ behaviors over time. To model these changes, we scaled the contribution of each contact matrix for location x,

$$r_{m}=\underset{x}{\sum }\alpha_{m,x}r_{x}.$$ [1]

The weights $\alpha_{m,x}$ depend on disease and symptom status (m) and location (x) as specified in Table 2. We scaled social contacts for symptomatic individuals following changes in behavior observed among symptomatic individuals during the 2009 A/H1N1 pandemic (30). For those without symptoms (susceptible and asymptomatic), the weights were specified to match reduced levels of social contacts as the product of social distancing policies. Home contact rates were held constant, and nonhousehold contact rates were roughly based on survey data from ref. 15. However, levels of social distancing have varied strongly over time and between locations. To account for this variability, we tested a range of alternative levels in addition to the base model. The results for these alternative parameter values are discussed in *SI Appendix*, section A. Also, notably, we do not consider the seasonality of contact rates for children in the scenarios where schools are modeled as closed. This would likely have limited impact on the optimal solutions, but, when this is not the case, we may overestimate or underestimate the importance of school contacts, depending on the time of year when vaccines are distributed.

**Table 2.** Weights on contact rates for a given disease and symptom type (m) and location/activity (x) under social distancing

	Contact rate weights, $\alpha_{m,x}$			
Disease and symptom type	Home	Work	School	Other
Symptomatic	1.0	0.036	0.036	0.075
Susceptible or asymptomatic	1.0	0.4*, 0.1	0.3	0.4

When essential and nonessential worker weights are both needed, the former is marked with an asterisk.

The proportion of essential workers in the population was set to be consistent with estimates of the portion of jobs that can be done from home (31) and estimates from the US Cyber-security and Infrastructure Security Agency, which indicate that 70% of the workforce is involved in these essential activities (e.g., healthcare, telecommunications, information technology systems, defense, food and agriculture, transportation and logistics, energy, water, public works, and public safety) (32). However, essential workers are not a cleanly defined group of individuals, and there is heterogeneity in the level of contact rates within this group. As a robustness check on this base scenario approach, we also tested a model with a smaller number of essential workers with higher contact rates. Results from this model are discussed in *SI Appendix*, section D.

### Transmission Rate and Vaccine Effectiveness.

The model was calibrated to match the predicted $R_{0}$ for COVID-19 in the United States (see *SI Appendix*, Table S1) by solving for probability of transmission q, assuming a naive (prepandemic) population. Details of this procedure are provided in *SI Appendix*, section A.

In our base model, we considered vaccine effectiveness of 90%. This level is at the low end of the range of estimates reported (90 to 95%) for reduction in symptomatic infections in the fall of 2020 from phase three clinical trials (33). We selected the low end, since real-world performance is typically somewhat lower than clinical trial effectiveness, for example, due to imperfect implementation of dual-dose timetables and/or cold storage requirements. We also assume this effectiveness is the same across age groups, since initial evidence does not show substantial differences between subgroups (34). As an alternative, lower-bound scenario, we considered vaccine effectiveness of 50%, since this is the minimum expectation of the US Food and Drug Administration (FDA) for approval (35). Finally, we considered a case where the vaccine is less effective for ages 60+ y. The phase three trials do not fully resolve the effectiveness of the vaccines by age, leading to uncertainty. This scenario represents a worst-case scenario where the vaccine is much less efficacious for the most sensitive groups.

### Initial Conditions.

Because the expected epidemiological conditions $\left\{I_{pre}\left(0\right),I_{asym}\left(0\right),I_{sym}\left(0\right),S\left(0\right)\right\}$, by the time the initial vaccine doses are ready for deployment, are uncertain, we consider a range of possible values from 1 case per thousand to 20 cases per thousand. These cases were apportioned between demographic groups to reflect the attack rates of COVID-19 for each group under the given social distancing policy. Alternative levels considered for initial conditions are described in *SI Appendix*, section A and appear in *Results* (Table 1 and Figs. 4 and 5 *C*).

### Vaccine Prioritization Optimization.

The planner’s decision problem is to allocate the daily supply of vaccine (v(t)) across the demographic groups according to a given objective. We assume that this group allocation vector, μ, can be chosen on a monthly basis at the beginning of each of the first six decision periods. We also assume that only susceptible individuals are vaccinated. We numerically solved for vaccine allocation strategies that minimize the total burden associated with three different health metrics: deaths, YLL, or symptomatic infections,

$$\text{deaths:}\min\left\{\overset{T}{\underset{0}{\int }}\underset{i\in J}{\sum }I_{sym,i}\left(t\right)/\gamma_{sym}dt\right\}$$ [2]

$$\text{YLL:}\min\left\{\overset{T}{\underset{0}{\int }}\underset{i\in J}{\sum }e_{i}\delta_{i}I_{sym,i}\left(t\right)/\gamma_{sym}dt\right\}$$ [3]

$$\text{symptomatic infections:}\min\left\{\overset{T}{\underset{0}{\int }}\underset{i\in J}{\sum }\delta_{i}I_{sym,i}\left(t\right)/\gamma_{sym}dt\right\},$$ [4]

where $e_{i}$ is the years remaining of life expectancy for group i and with a 6-mo time horizon (T=180 d). Preventing deaths and YLL are “consensus value(s) across expert reports” (ref. 4, p. 2052), while some argue that “protecting public health during the COVID-19 pandemic requires … minimizing COVID-19 infection” (ref. 5, p. 10).

We solved for the optimal allocation of available vaccines across demographic groups for each month over 6 mo. We identified the optimal solution using a two-step algorithm. In the first step, we used a genetic algorithm similar to ref. 36 to identify an approximate solution. This approach uses random sampling of the potential solution space to broadly explore, in order to avoid narrowing to a local and not global minimum. In the second step, we used simulated annealing to identify the solution with precision. At a given optimal solution, it may or may not be the case that the outcome of interest (e.g., minimizing deaths) is sensitive to small changes in the allocation decision. Thus, around the optimal allocation, we also identified nearby allocations that produce outcomes that are less desirable but still within 0.5% of the optimized outcome. A detailed description of the algorithm is given in *SI Appendix*, section A. All code for the optimization was written in the Julia programming language (37).

The whiskers on optimal vaccine allocation bars in Fig. 2 show the range of alternative allocations that still produce an outcome that is within 0.5% of the optimum. The upper (lower) bound of each whisker was produced one at a time by systematically exploring higher (lower) levels of the given decision variable (proportion of vaccines allocated to a given demographic group in a given decision period). This entailed fixing a candidate level of that decision variable as a constraint and optimizing the remaining parameters. If the optimized value of this constrained objective function was within 0.5% of the unconstrained optimum, then the candidate value was accepted and included within the bounds specified by the whisker. The whisker bounds were found using bisection linear search algorithm tuned to identify each bound to within one percentage point of the true value (see *SI Appendix*, section A).

To assess the benefits of 1) using a dynamic allocation policy and 2) differentiating by essential worker status in addition to age, we constructed two constrained policies: a static policy and an age-only policy. The static policy was found by allowing the proportion of vaccine allocated to each age group to be chosen once when the vaccine first becomes available and then applied constantly over time.** The age-only policy simply involves constraining allocation choices age groups (not differentiated by essential worker status)—vaccines allocated to working age groups accrue to essential workers simply in proportion to their relative share of these groups.

### Data Access.

All data used for informing the numerical analysis are freely available at the source noted for each measure. The data and code used to initialize and run the models is available on Github, https://github.com/JackBucknerNRM/Vaccine_prioritization.

## Supplementary Material

Supplementary File

## Data Availability

Code and parameter files for model analysis have been deposited in Github (https://github.com/JackBucknerNRM/Vaccine_prioritization).

## Footnotes

^†^We also note that ref. 18 uses simulation without optimization to explore implications of vaccines with various levels of direct and indirect protection.

^‡^We do not discount in our calculation of YLL; doing so would lead to more equal weighting on mortality across age groups and thus results that are closer to those when minimizing deaths.

^§^Excess vaccine is allocated without targeting if all of the susceptible individuals in a given group have already been vaccinated.

^¶^See resulting YLL and resulting infections for the deaths objective with age-only and static constraints.

^#^In the event that vaccination requires two doses over time, we consider an individual vaccinated upon receipt of the second dose at time t, and we assume that v indicates the number of individuals that can be vaccinated with the required number of doses.

^∥^This vaccine effectiveness is inclusive of any efficiency loss from typical handling in the distribution chain.

**If all of the susceptibles from a single group were exhausted (either by full coverage from the vaccine or from infection), then vaccine that would have been allocated to individuals from that group are instead allocated to other age groups at a rate proportional to the size of their susceptible population.
