# Supplementary material for: Dynamic prioritization of COVID-19 vaccines when social distancing is limited for essential workers
Source: Proc Natl Acad Sci U S A. 2021 Apr 2;118(16):e2025786118. doi: 10.1073/pnas.2025786118 (PMC8072365; doi:10.1073/pnas.2025786118)
Supplement: Supplementary File [file pnas.2025786118.sapp.pdf]

Dynamic Prioritization of COVID-19 Vaccines When Social Distancing is Limited for Essential Workers: Supplemental Information

Appendix

A Model specification, parameterization and optimization

A.1 Model dynamic equations

The dynamic equations specifying transitions between the disease states are as follows:

$$\dot{S}_i = -qs_i\theta \left[\sum_{j \in J} \sum_{m \in M} \tau_m r_{m,i,j} S_i \frac{I_{m,j}}{N_j} \right] - \mu_i v \tag{1}$$

$$\dot{F}_i = -qs_i\theta \left[\sum_{j \in J} \sum_{m \in M} \tau_m r_{m,i,j} F_i \frac{I_{m,j}}{N_j} \right] + (1 - \epsilon_i)\mu_i v \tag{2}$$

$$\dot{E}_i = qs_i\theta \left[\sum_{j \in J} \sum_{m \in M} \tau_m r_{m,i,j} (S_i + F_i) \frac{I_{m,j}}{N_j} \right] - E_i/\gamma_{exp} \tag{3}$$

$$\dot{P}_i = \epsilon_i \mu_i v \tag{4}$$

$$\dot{I}_{pre,i} = E_i/\gamma_{exp} - I_{pre,i}/\gamma_{pre} \tag{5}$$

$$\dot{I}_{asym,i} = \sigma_{asym} I_{pre,i}/\gamma_{pre} - I_{asym,i}/\gamma_{asym} \tag{6}$$

$$\dot{I}_{sym,i} = (1 - \sigma_{asym}) I_{pre,i}/\gamma_{pre} - I_{sym,i}/\gamma_{sym} \tag{7}$$

$$\dot{R}_i = I_{asym,i}/\gamma_{asym} + (1 - \delta_i) I_{sym,i}/\gamma_{sym} \tag{8}$$

$$\dot{\gamma}_i = \delta_i I_{sym,i}/\gamma_{sym}. \tag{9}$$

To reduce clutter we have suppressed the time index t on each of the state variables, the vaccine allocation vector μ_i, and the vaccination rate v.

A.2 Model parameters

Parameter	Description	Base Value(s)	Source
J	demographic groups: (1) age-only,<br>(2) age and essential workers	{0-4, 5-19, 20-39, 40-59, 60-74, 75+},<br>{0-4, 5-19, 20-39, 20-39*, 40-59, 40-59*, 60-74, 75+}	Assumed
σ_{asym}	infection asymptomatic rate	0.16	(1)
δ	infection fatality rate (age-specific)	{6.7×10^{-6}, 2.5×10^{-5}, 0.0002, 0.002, 0.018, 0.12}	(2)
s	susceptibility (age-specific)	{0.5, 0.5, 1.0, 1.0, 1.0, 1.0}	(3)
τ_{pre}<br>τ_{asym}<br>τ_{sym}	relative infectiousness by symptom type	0.51<br>0.51<br>1.0	(4)
γ_{exp}<br>γ_{pre}<br>γ_{asym}<br>γ_{sym}	symptom duration (days)	3.0<br>3.2<br>3.5<br>7.0	(4)
ϵ	vaccine effectiveness	0.9	Informed by initial COVID-19 vaccine effectiveness estimates (5)
p	proportion of essential workers	0.40	Calculated with labor data (6, 7); alternative: (8)
R_0	secondary infections in a naive population	2.5	(9), (10)
q	transmission probability in a naive population	0.053	Calculated given R_0, s and other parameters
θ	scaling factor for transmission probability due to NPI other than social distancing	0.65	Assumed (consistent with estimated COVID-19 R_0 under NPIs (10))
n	population shares: (1) age-only,<br>(2) age and essential workers	{0.06, 0.19, 0.27, 0.26, 0.19, 0.04},<br>{0.06, 0.19, 0.19, 0.08, 0.18, 0.8, 0.19, 0.04}	(11)
e	remaining years of life expectancy (age-specific)	{76, 66, 50, 31, 17, 6}	(12)
$R(0)$<br>$I_{sym}(0) + I_{asym}(0)$	initial recovered<br>initial sympt. and asympt.	0.08<br>0.005	Informed by IHME projections (13)
v	fraction of population vaccinated daily	0.1/30	Informed by comments from CDC Director to U.S. Senate Panel (14)

Table S.1: Base model parameter values and sources.

A.3 Parameters for alternative scenarios

Scenario	Change from Base scenario parameters	Source
High initial infections	15 symptomatic infections per 1000	Assumed
Strong NPI	$\theta = 0.5$	Assumed
Weak NPI	$\theta = 0.75$	consistent with 30-70% of U.S. population always wearing a mask (15) with 33-58% effectiveness (16)
Weak vaccine	$\epsilon_i \in \{0.5, 0.5, 0.5, 0.5, 0.5\}$	Minimum value from FDA approval
Weak vaccine seniors	$\epsilon_i \in \{0.9, 0.9, 0.9, 0.9, 0.5, 0.5\}$	Informed by influenza vaccine effectiveness
High susceptibility ages < 20	$s_i \in \{1.0, 1.0, 1.0, 1.0, 1.0\}$	Assumed
Low supply	$v(t) = 0.05/30$<br>allocation policies switched every 60 days	Assumed
Ramp up	$v(t) = \left\{ \begin{array}{ll} 0.05/30, & t \leq 60 \\ 0.10/30, & t > 60 \end{array} \right]$<br>Allocation polices switch for every 10% of population vaccinated	Informed by comments from the scientific head of the U.S. vaccine development program (17)
Open schools	$\alpha_{school} = 0.7$	Assumed
High contacts	$\alpha_{social} = 0.5$	Assumed

Table S.2: Parameter values that differ from the Base case for alternative scenarios.

A.4 Initial conditions

The number of susceptible, infected and recovered individuals is likely to vary by region and will depend on the time when the vaccine is deployed. Because of the likely variation in this parameter, we test a range of values from 1-20 symptomatic cases per 1000 (when the vaccine is deployed). The infections are assumed to be distributed between groups in accordance with the stable distribution of cases when the epidemic is growing exponentially. The portion of each group infected at time $t = 0$ in the Base parameter set is shown in Fig. S.1. The number of recovered individuals in the population was set to 8% of each demographic group which was informed by IHME projections (13). When the vaccine is first deployed, we set the number of deceased and vaccinated individuals in each age group to zero (since the share of actual deceased is very small, i.e. approximately 0.0015). The number susceptible was set to ensure the proportion of individuals in each category summed to 100% after the number of individuals at each stage of infection was determined.

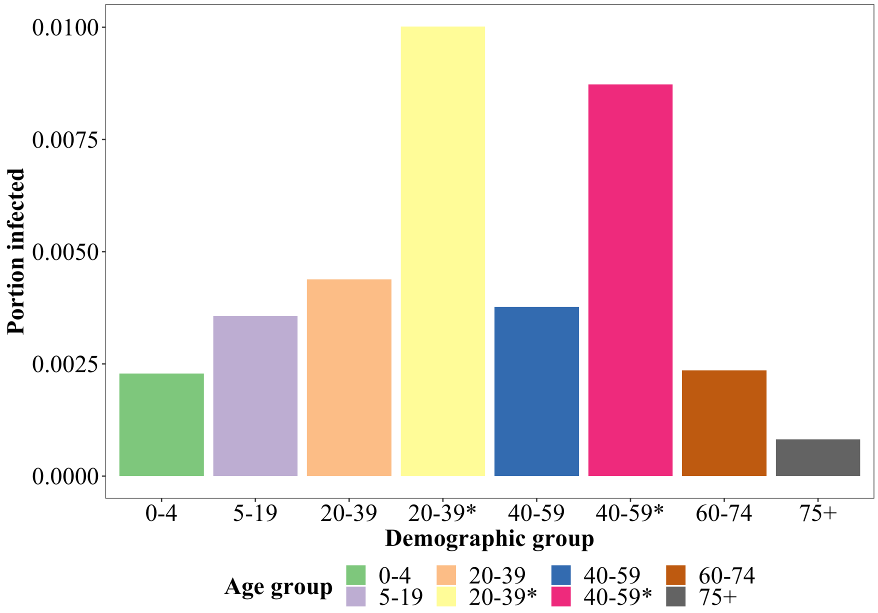

Figure S.1: The proportion of each demographic group infected when vaccine distribution begins in the Base scenario.

A.5 Calibration

The relationship between the basic reproduction number, R_0, and parameters governing transmission and epidemiological characteristics is given by the so-called next-generation matrix:

$$R_0 = max[eigenval\{q\gamma_{pre}\tau_{pre}(r\Delta s)\Delta n + q\sigma_{asym}\gamma_{asym}\tau_{asym}(r\Delta s)\Delta n + q(1-\sigma_{asym})\gamma_{sym}\tau_{sym}(r\Delta s)\Delta n\}], \tag{10}$$

where the maximum eigenvalue operator wraps several terms including r, the social contact matrix, s, the age-specific susceptibility rate, n, a vector of the proportions of the population in each demographic group and Δ, an operator that signifies multiplying each row of a matrix by the corresponding entry in the vector. For symptom type $m \in \{asym, pre, sym\}$, the constants γ_m, τ_m and σ_m represent the duration, relative infectiousness of an individual and the probability of type m, respectively.

We first set a baseline $R_0 = 2.5$ as estimated by (9). We then solve for the transmission probability parameter, q, using Equation 10, assuming a naive (pre-pandemic) population. We then scaled q by a fixed factor $\theta \in [0, 1]$ to reflect the impact of non-pharmaceutical interventions (NPI) like masks, hand washing and maintaining distance when contacts are made.

A.6 Contact matrices distinguishing essential workers

Estimated contact rates for the U.S. were obtained from (18) who used population-based contact diaries from the European POLYMOD survey to project to other countries, including the U.S. These included contact rates for 16 age classes in five year increments from ages 0 to 80. We collapsed these to five age groups (0-4, 5-19, 20-39, 40-59, 60-80) using population-weighted sums:

$$\hat{r}_{i,j,x} = \sum_{\mathtt{i}\in i}\left(\frac{N^{pop}_{\mathtt{i}}}{N^{pop}_{i}}\sum_{\mathtt{j}\in j} r_{\mathtt{i},\mathtt{j},x}\right), \tag{11}$$

where $\{\mathtt{i},\mathtt{j}\}$ are the subscripts for the five year age bins, $\{i,j\}$ are the subscripts for the larger age bins, $r_{\mathtt{i},\mathtt{j},x}$ is the average number of daily contacts a person in group $\mathtt{i}$ makes with a person in group $\mathtt{j}$ for activity/location x, and N^{pop}_h is the population size for age group h.

The total number of i-to-j contacts must equal the total number of j-to-i contacts: $N^{pop}_i r_{i,j,x} = N^{pop}_j r_{j,i,x}$. Because numerical issues—estimation in (18), bin discretization and rounding—can lead to small differences, we ensure this condition holds by imposing,

$$r_{i,j,x} = \frac{0.5(N^{pop}_i \hat{r}_{i,j,x} + N^{pop}_j \hat{r}_{j,i,x})}{N^{pop}_i}, \tag{12}$$

where the numerator is the mean of the two measures of total contacts between groups i and j and the denominator transforms the result to per-capita in i.

Setting essential worker contact rates requires additional assumptions and attention to the activity/location. We define the essential worker indicators $e \in \{n, y\}$ for "no" and "yes". Our grouping is such that all essential workers ($e = y$) are employed but non-essential-workers ($e = n$) are a mix of employed and not employed. Let e' represent the indicator for a second group which can be equal or not equal to the value for e.

In the case of all activities/locations x that are not *work*, contact rates are given by

$$r_{(i,e),(j,e'),x} = \frac{N^{pop}_{(j,e')}}{N^{pop}_j} r_{i,j,x}, \ \ \forall\, x \neq work. \tag{13}$$

This follows from the assumption that contacts made by any group (i, e) with any other group (j, e') are independent of i's essential worker status. Thus, we only need to split contacts $r_{i,j,x}$ into those made with essential worker type $e' = y$ versus the remainder with type $e' = n$, i.e. given the share $N^{pop}_{(j,e')}/N^{pop}_j$.

Estimating contacts when $x = work$ involves a larger number of steps. We first address contacts made by essential workers ($e = y$) before turning to non-essential workers ($e = n$). For $e = y$, let the share of the working age population $(20 - 59)$ in group i that is employed be given by p_i.

We assume that all of the work contacts are attributable to employed adults resulting in an employed adult contact rate of $r_{i,j,work}/p_i$. Then the contact rate of essential workers ($e = y$) in group i with age group j is

$$r_{(i,y),j,work} = \frac{r_{i,j,work}}{p_i}. \tag{14}$$

Let the fraction of working age group i that is employed in an essential worker role be given by $p_{i,y}$. The average workplace contact rate for non-essential-workers in group i with group j is given by

$$r_{(i,n),j,work} = \alpha_{work}\left(\frac{r_{i,j,work}}{p_i}\right)\left(\frac{p_i - p_{i,y}}{1 - p_{i,y}}\right), \tag{15}$$

where $\alpha_{work} < 1$ scales for social distancing and the final term in brackets scales for the share of non-essential workers that are employed and thus have contacts at *work*.

Finally, we assume that the average *work*place contact rate for an individual of type (i, e) with individuals of type (j, e') is given by the partial contact rate $r_{(i,e),j,work}$ times the proportion of total work contacts of individuals in group j that are made by individuals in sub group e':

$$r_{(i,e),(j,e'),work} = r_{(i,e),j,work}\left(\frac{N_{(j,e')}\cdot r_{i,(j,e'),work}}{N_{(j,y)}\cdot r_{i,(j,y),work}+N_{(j,n)}\cdot r_{i,(j,n),work}}\right). \tag{16}$$

In addition to The formulation described above we also considered a scenario where essential workers contacts were clustered (i.e. individuals only contact others of the same essential worker status at work). The work contact rates for each group are calculated in the same manner as described above, but we assume that the c work contacts between essential and non-essential workers are zero.

These two model formulation represent two extremes. Work contacts are likely to be concentrated among others of the same essential status (as opposed to formulation one) but essential workers are likely to have some contacts non-essential workers in the work place.

Finally we scale the work contacts for age groups that are not separated into essential and non-essential workers (5-19, 60-80) to match the scaling for prime working age classes.

$$r_{i,j,work} = \left(\frac{p_{i,y}}{p_{work}}+\alpha\frac{p_{work}-p_{i,y}}{p_{work}}\right)r_{i,j,work} \tag{17}$$

A.7 Optimization algorithm

The optimization algorithm used in our analysis is split into two parts. First a genetic algorithm is run to identify an effective strategy near a global optimum. This solution is then refined via simulated annealing.

Genetic algorithms take inspiration from the natural process of evolution, and work by randomly sampling a populating of candidate solutions, selecting a set of survivors based on the candidates performance against the objective function, information from these survivors is then used to generate a new generation of candidates solutions, and so forth (19). The genetic algorithm executes the following steps:

1. Sample $N_{t=0}$ candidate solutions $\{x_{n,t=0}\}$ from a Dirichlet distribution with parameter α_0.

2. Each candidate solution is evaluated with the objective function.

3. The bests $K_{t=0}$ candidates $\{x^{best}_{n,t=0}\}$ are solved and the distributions parameter α_0 is updated to α_1 which is the mean of $\{x^{best}_{n,t=0}\}$ times the entropy parameter for that time step η_t. The entropy parameter determines how concentrated new samples will be around the mean of the selected samples in the prior step.

4. Steps 1 to 3 are repeated for a fixed number of iterations T and the best candidate solution sampled at any iteration is returned. The values N_t, K_t and η_t are tuned for each step to maximize performance.

Simulated annealing is based on thermodynamic models of cooling metals. Briefly, the algorithm is initialized by sampling a candidate solution x_0, this candidate solution is updated by sampling a new candidate solution x_t from a proposal distribution centered around x_0. This solution is either accepted and replaces the current x_0 or it is rejected and a new candidate solution is drawn using the existing value of x_0. The proposed solutions x_t are accepted if they perform better against the objective than the incumbent x_0, if $x_t > x_0$ it is selected with probability $p = exp\left[-(x_t - x_0)/T_t\right]$. large values of T_t increase the probability that a new candidate solution will be accepted allowing the algorithm to explore the solution space and move away from local minima. T_t is reduced over time to allow the algorithm to start exploring the solution space and then eventually stabilize on a global minimum. The simulated annealing executes the following steps:

1. Initialize a chain with value x_0. Generate a new sample from the proposal distribution $x_t \sim tr(N(tr^{-1}(x_0), \sigma I))$ where the transform tr from $\mathbf{R}^n$ to the solution space. Initialize a counter i that tracks the number of iterations.

2. If $x_t < x_0$ replace x_0 with x_t, update $i = i + 1$ and repeat from step 1.

3. If $x_t > x_0$ sample $\mu \sim unif(0, 1)$. If $\mu > exp\left(-(x_t - x_0)/T(i)\right)$ then replace x_0 with x_t update i and repeat from step 1. Otherwise save x_0 and repeat from step 1. We used $T(i) = T_0/i$ as the temperature function.

4. Stop when $i > max\_iter$

These algorithms were tuned experimentally to consistently converge on a minimum solution on a test case. We used the minimum years of life lost under the Base parameter set as our test case. We found that numerical errors (defined as difference between the test runs) increased over the decision periods. This is caused by the face that the final decision periods are a region of very flat payoff, leading to a large number of solutions that perform very similarly but differ in this region

To quantify the sensitivity of the solutions to deviations in the outcome of interest, for each decision variable, we identified the range of alternative values nearby that produced a similar objective function value. This procedure is described in Appendix A.8.

A.8 Whiskers on optimal vaccine allocation bars in Fig. 2

The whiskers on optimal vaccine allocation bars in Fig. 2 show the range of alternative allocations that still produce an outcome that is within 0.5% of the optimum. The upper (lower) bound of each whisker was produced one at a time by systematically exploring higher (lower) levels of the given decision variable (proportion of vaccines allocated to a given demographic group in a given decision period). Let x represent the level of a single decision variable. The whiskers for x were found in a two step process. First, approximate upper (lower) bounds on the range of x were found by sampling new candidate solutions above (below) the optimum with a Markov chain using the algorithm below.

1. Initialize a chain with the optimized value $x_0 = x^*$. Generate a new sample from the proposal distribution $tr(x_t) \sim N(tr^{-1}(x_0), \sigma I)$, where the function applies the soft max function to each decision period so that the solutions are represented by a vector in $R^{(n_{groups}-1)*n_{steps}}$, I is the identity matrix and $\sigma = 0.001$. $n_{groups} = 8$ is the number of demographic groups and $n_{steps} = 6$ is the number of decision periods. Initialize a counter i that tracks the number of iterations.

2. If $f(x_i) < 1.005 * f(x^*)$, where f returns the value of the objective function, replace x_{i-1} with x_i, update $i = i + 1$, save x_i and sample a new candidate solution centered at x_i.

3. Else reject x_i and sample a new candidate solution centered at x_{i-1}.

4. Stop when $i > N_{samples} = 10000$

5. Repeat steps 1-4 to generate samples from $N_{chains} = 30$ independent chains. For each decision variable x, return the maximum ($\bar{x}$) and minimum ($\underline{x}$) value from the list selected samples.

For each decision variable, this algorithm produces the pair ($\underline{x}$, $\bar{x}$), an upper bound for the minimum whisker value and, similarly, a lower bound for the maximum value for the whisker. We refined this solution as follows. For each decision variable, we take $\underline{x}$ and explore alternative candidate values $x^{cand} \in [0, \underline{x}]$, i.e., values between the lower bound on the decision variable and $\underline{x}$. To do so we use the bisection root finding algorithm adjusting the value of x^{cand} until $f(x^{cand}) - 1.005 * f(x^*) \leq tol = 0.01$. The procedure is repeated for the upper extent of the whisker for $x^{cand} \in [\bar{x}, 1]$. Thus the final whisker extents are each within a percentage point of the true bound.

B Static policies

We found that the patterns observed for our dynamic solutions were similar for the static policies. The static policies targeted the same high priority groups: ages 5-19 and essential workers when minimizing infections, and ages 60+ along with essential workers when minimizing deaths and YLL. Again consistent with the dynamic policies we found that optimal vaccine allocations were substituted towards essential workers when the effectiveness of the vaccine was low and when the reproductive number when the vaccine first became available was small. These patterns are shown in Fig. S.2A. [1]

[1] It should be noted that the quantities plotted in Fig. S.2A correspond to the allocation of the initial supply, which is different from the similar main text Fig. reffig:heatmap$_robustnessthatpresentsthepercentofeachgroupvaccinatedat3months.$

In Fig. S.2B we show how robust the static policies are when applied to the "wrong" scenario. As with the dynamic policies we found that most polices performed very poorly when applied to the weak vaccine ages 60+ and the strong NPI scenarios, relative to the optimum. But, in general, deterioration in performance (due to a mismatch between the true scenario and the one driving the policy applied) was much worse for static than dynamic polices. One driver of this effect is that static polices identify one set of high priority groups and do not switch. Dynamic polices also identify one set of high priority groups but then also switch. Thus dynamic policies differ by when a group is prioritized, as opposed to the static polices which differ by which groups are a high (unchanging) priority.

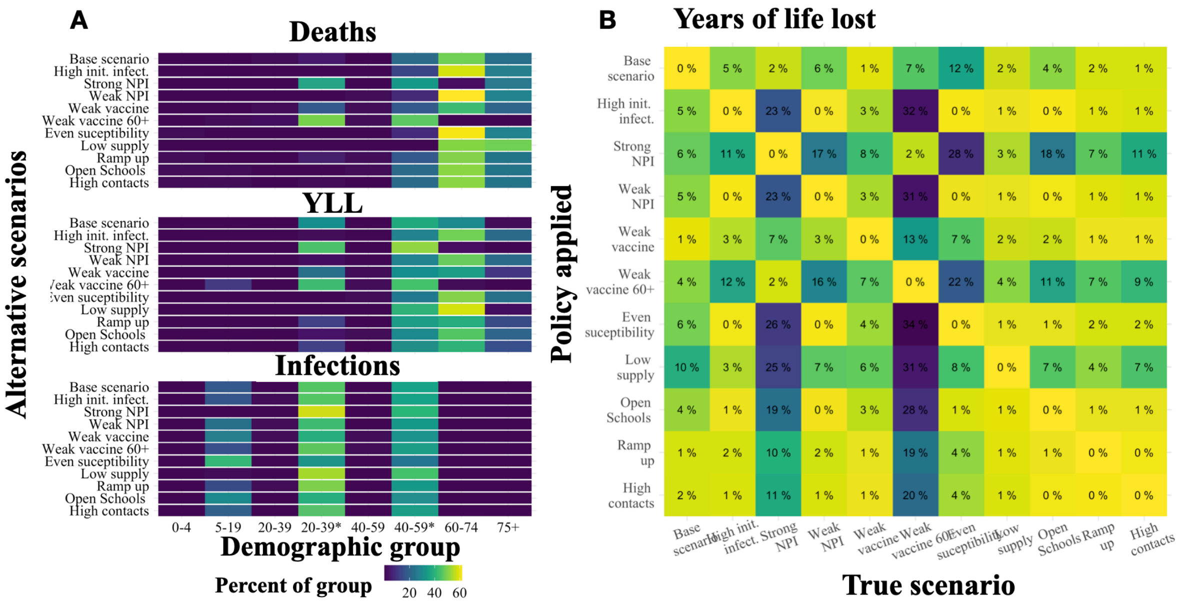

Figure S.2: The sensitivity of static policies to the alternative scenarios as given by the percent of the initial supply allocated to each demographic group (A), the performance relative to the optimum allocation of each policy when applied to each of the alternative scenarios when the objective is YLL (B).

C Additional model robustness results

Here we present performance loss due to a mismatch between the true scenario and the one used to establish the allocation policy. Results are presented for either minimizing deaths (Fig. S.3) or infections (Fig. S.4).

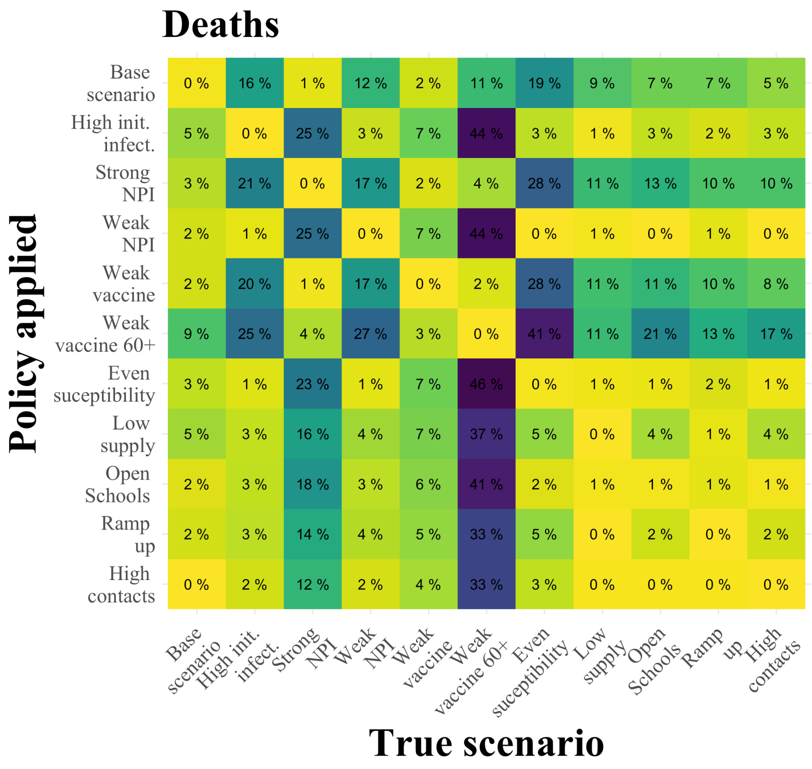

Figure S.3: The percentage of additional deaths in excess of the optimum when applying a policy for an alternative scenario (row) to an alternative "true" scenario (column).

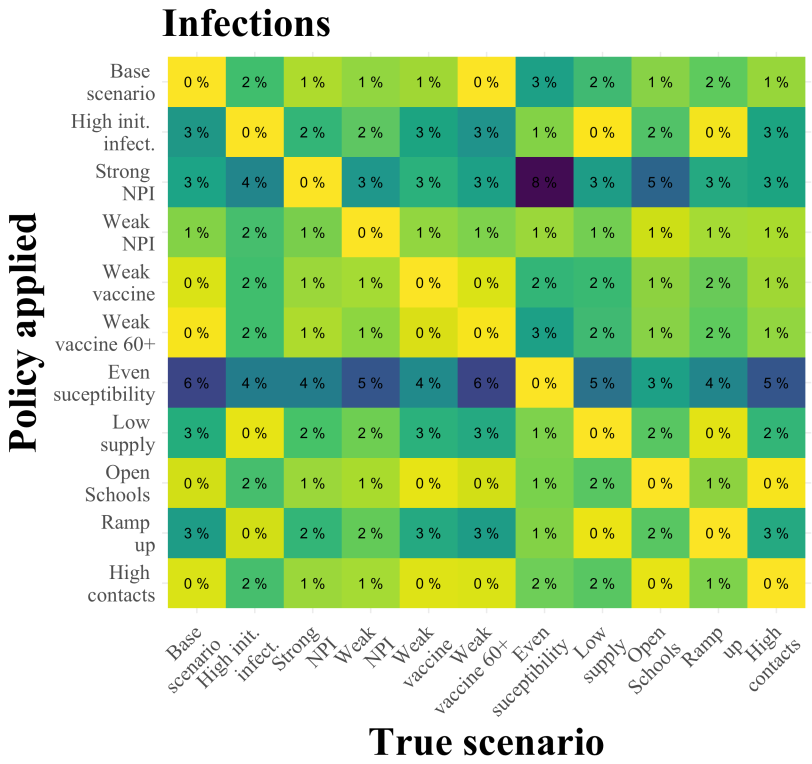

Figure S.4: The percentage of additional infections in excess of the optimum when applying a policy for an alternative scenario (row) to an alternative "true" scenario (column).

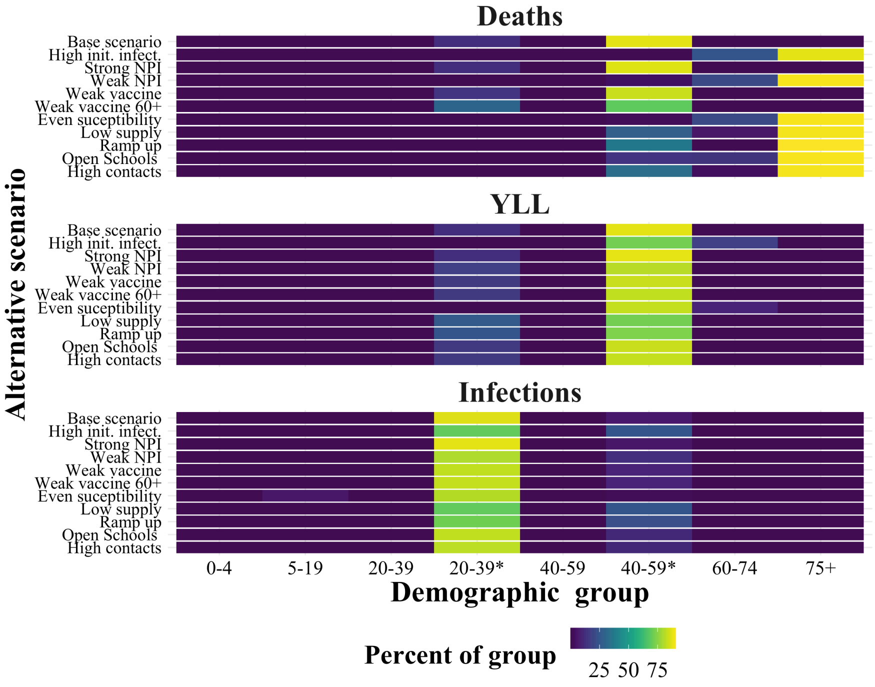

Figure S.5: The percentage of each demographic group vaccinated after the fist decision period under each objective and the alternative scenarios.

D Alternative model structures

In addition to considering a range of alternative parameter sets, we tested our results against three alternative model structures: (1) clustered essential workers: essential workers only contact other essential workers in the work place; (2) concentrated essential workers: 20% of the working age population are "essential" and have substantially higher (slightly above doubled) contact rates than essential workers in the Base model; and (3) leaky vaccine: the vaccine reduces the susceptibility of all vaccinated individuals to infection, and reduces their infectiousness and risk of death if infected. The corresponding parameter values for each alternative model are summarized below in Table S.3 and the model structure for the leaky vaccine model is given in SI Appendix D.1. Results presented in Fig. S.6 are discussed in the main text.

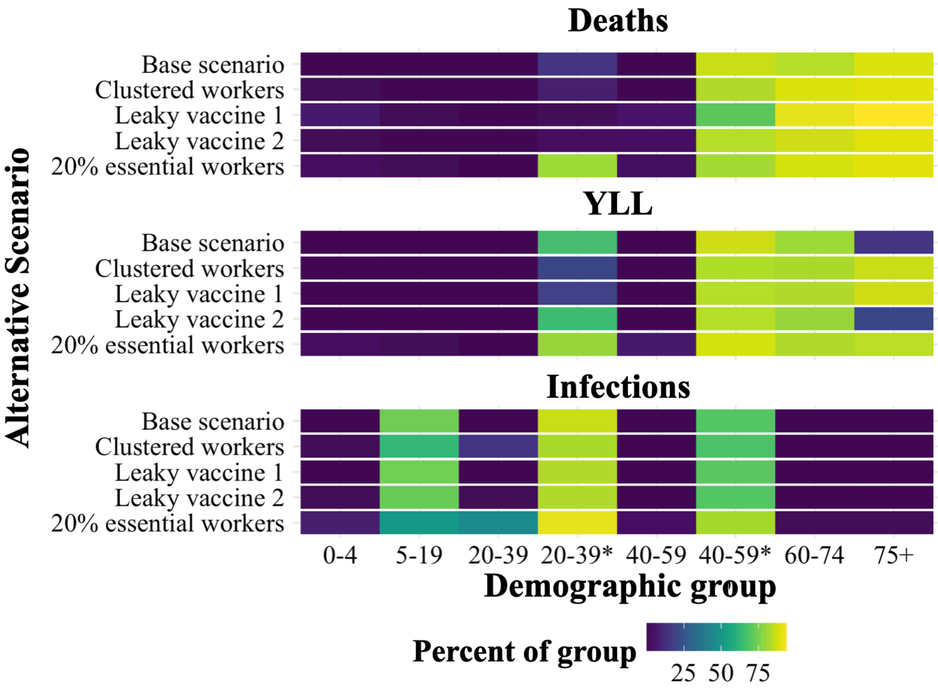

Figure S.6: The percent of each demographic group (horizontal axis) vaccinated after three months under the optimal policy for each of the alternative model structures (vertical axis) and objectives (panels).

Model	New parameters
Clustered essential workers	Essential workers contacts focused within group as discussed in SI Appendix A.6
Concentrated essential workers	The proportion of essential workers is 20% work; contact rates are increased for essential workers ($\alpha_{work} = 0.1$, $\alpha_{work}* = 1.0$)
Leaky vaccine 1	$VE_{sucpt} = VE_{trans} = VE_{sym} = 0.9$
Leaky vaccine 2	$VE_{sucpt} = 0.9$, $VE_{trans} = VE_{sym} = 0$

Table S.3: The parameter values changed between the alternative model structures and the base model.

D.1 Leaky vaccine specification

Vaccines can provide multiple forms of protections against infections. Among these protections is the ability for vaccines to prevent individuals from becoming infected (the case considered in the main text). In addition, if vaccinated individuals still become infected they may (1) be exhibit reduced infectiousness and/or (2) develop less severe symptoms. To allow for these latter two cases we changed the model structure to track vaccinated and infected individuals. To do this we maintained the protected and uninfected category P and added four categories: vaccinated and exposed class P_{exp}, vaccinated and pre-symptomatic P_{presym}, vaccinated and asymptomatic P_{asym} and vaccinated and symptomatic P_{sym}. The effectiveness of the vaccine is modeled with three age specific vectors, VE_{sucpt}, VE_{trans}, and VE_{sym}, which quantify the extent to which the vaccine reduces the susceptibility of vaccinated individuals to infection, the reduction in infectiousness of vaccinated individuals and the reduction in infection fatality rate of vaccinated individuals. This new model can be described by the following system of equations:

$$\dot{S_i} = -qs_i\theta\left[\sum_{j\in J}\sum_{m\in M}\tau_m r_{m,i,j}S_i\frac{I_{m,j}}{N_j} + \tau_m r_{m,i,j}S_i\frac{P_{m,j}(1-VE_{trans})}{N_j}\right] - \mu_i v \tag{18}$$

$$\dot{P_i} = -qs_i(1-VE_{sucpt,i})\theta\left[\sum_{j\in J}\sum_{m\in M}\tau_m r_{m,i,j}P_i\frac{I_{m,j}}{N_j} + \tau_m r_{m,i,j}P_i\frac{P_{m,j}(1-VE_{trans})}{N_j}\right] + \mu_i v \tag{19}$$

$$\dot{E_i} = qs_i\theta\left[\sum_{j\in J}\sum_{m\in M}\tau_m r_{m,i,j}S_i\frac{I_{m,j}}{N_j} + \tau_m r_{m,i,j}S_i\frac{P_{m,j}(1-VE_{trans})}{N_j}\right] - E_i/\gamma_{exp} \tag{20}$$

$$\dot{P_{exp,i}} = qs_i(1-VE_{sucpt,i})\theta\left[\sum_{j\in J}\sum_{m\in M}\tau_m r_{m,i,j}P_i\frac{I_{m,j}}{N_j} + \tau_m r_{m,i,j}P_i\frac{P_{m,j}(1-VE_{trans})}{N_j}\right] - E_i/\gamma_{exp} \tag{21}$$

$$\dot{I}_{pre,i} = E_i/\gamma_{exp} - I_{pre,i}/\gamma_{pre} \tag{22}$$

$$\dot{P}_{pre,i} = P_{exp_i}/\gamma_{exp} - P_{pre,i}/\gamma_{pre} \tag{23}$$

$$\dot{I}_{asym,i} = \sigma_{asym}I_{pre,i}/\gamma_{pre} - I_{asym,i}/\gamma_{asym} \tag{24}$$

$$\dot{P}_{asym,i} = \sigma_{asym}P_{pre,i}/\gamma_{pre} - P_{asym,i}/\gamma_{asym} \tag{25}$$

$$\dot{I}_{sym,i} = (1-\sigma_{asym})I_{pre,i}/\gamma_{pre} - I_{sym,i}/\gamma_{sym} \tag{26}$$

$$\dot{P}_{sym,i} = (1-\sigma_{asym})P_{pre,i}/\gamma_{pre} - P_{sym,i}/\gamma_{sym} \tag{27}$$

$$\dot{R_i} = I_{asym,i}/\gamma_{asym} + (1-\delta_i)I_{sym,i}/\gamma_{sym} + (1-\delta_i\,(1-VE_{sym}))P_{sym,i}/\gamma_{sym} \tag{28}$$

$$\dot{\gamma_i} = \delta_i(1-VE_{sym})P_{sym,i}/\gamma_{sym} + \delta_i(1-VE_{sym})I_{sym,i}/\gamma_{sym} \tag{29}$$

We consider two cases: a vaccine with equal effectiveness set to 90% for consistency with the Base model $VE_{sym} = VE_{sucpt} = VE_{trans} = 90\%$, and a vaccine that only reduces susceptibility to infections $VE_{sym} = 90\%$ and $VE_{sucpt} = VE_{trans} = 0\%$.

D.2 Contact rates sensitivity

One key source of both uncertainty and heterogeneity between communities is the true set of underlying social contact rates. To test the effects of these parameters we considered a range of work and other contact rates around those specified in the Base scenario. The "other" (outside of the home, school and workplace) contacts were scaled from the pre-COVID-19 average while the work contact rates were increased for essential workers and held at 10% of pre-COVID-19 levels for non-essential workers. Results are presented in Fig. S.7, where each scenario is labeled with the percentage change in average work contact rate and the contact rates of essential workers are a percentage of pre-COVID-19 average levels. We found that the policies did change as both other and work contacts increased and that these changes were consistent with the changes in reproductive number induced by the higher contact rates.

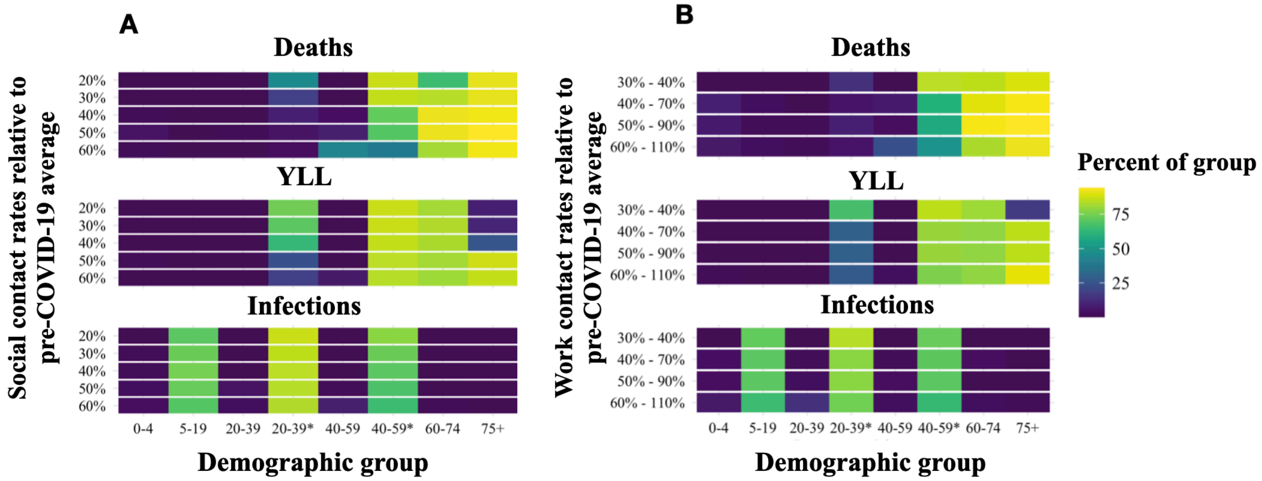

Figure S.7: The percentage of each demographic group vaccinated after 3 months under the optimal dynamic policy given variation in (A) "other" (non-work) contacts and (B) work contacts.

References

[1] O Byambasuren, et al., Estimating the extent of true asymptomatic COVID-19 and its potential for community transmission: systematic review and meta-analysis, Available at SSRN 3586675: `https://ssrn.com/abstract=3586675` (2020).

[2] AT Levin, KB Cochran, SP Walsh, Assessing the age specificity of infection fatality rates for COVID-19: Meta-analysis & public policy implications, National Bureau of Economic Research Working Paper No. 27597 (2020).

[3] NG Davies, et al., Age-dependent effects in the transmission and control of COVID-19 epidemics. *Nature Medicine* **26**, 1205–1211 (2020).

[4] S Abrams, et al., Modeling the early phase of the Belgian COVID-19 epidemic using a stochastic compartmental model and studying its implied future trajectories, (medRxiv, doi: 10.1101/2020.06.29.20142851), preprint (2020).

[5] M Herper, D Garde, Moderna to submit Covid-19 vaccine to FDA as full results show 94% efficacy (STAT, `https://www.statnews.com/2020/11/30/moderna-covid-19-vaccine-full-results/`) (2020).

[6] AW Bartik, ZB Cullen, EL Glaeser, M Luca, CT Stanton, What jobs are being done at home during the COVID-19 crisis? Evidence from firm-level surveys, National Bureau of Economic Research Working Paper No. 27422 (2020).

[7] LMI and C2ER, SOC codes for CISA critical infrastructure workers (2020) data retrieved from LMI institute webstite, `https://www.lmiontheweb.org/more-than-half-of-u-s-workers-in-critical-occupations-in-the-fight-against-covid-19/`.

[8] G McCormack, C Avery, AKL Spitzer, A Chandra, Economic vulnerability of households with essential workers. *JAMA* **324**, 388–390 (2020).

[9] AJ Kucharski, et al., Early dynamics of transmission and control of COVID-19: a mathematical modelling study. *The Lancet Infectious Diseases* **20**, 553 – 558 (2020).

[10] HM Korevaar, et al., Quantifying the impact of US state non-pharmaceutical interventions on COVID-19 transmission, (medRxiv, doi: 10.1101/2020.06.30.20142877), preprint (2020).

[11] Population Pyramid, Population of the United States (`https://www.populationpyramid.net`, accessed July 12, 2020) (2020).

[12] U.S. Social Security Administration (USSSA), Life Table (2020) Data retrieved from USSSA website, `https://www.ssa.gov/oact/STATS/table4c6.html`.

[13] Institute for Health Metrics and Evaluation (IHME), Covid-19 mortality, infection, testing, hospital

resource use, and social distancing projections (August 21, 2020 estimates) (Seattle, United States of America: Institute for Health Metrics and Evaluation (IHME), University of Washington, https://ihmecovid19storage.blob.core.windows.net/latest/ihme-covid19.zip) (2020).

[14] B Lovelace Jr., N Higgins-Dunn, CDC says U.S. should have enough coronavirus vaccine to return to "'regular life"' by third quarter of 2021 (cnbc.com) (2020).

[15] Premise, How mask-wearing has changed in America: A visual journey of increasing mask use (San Francisco, United States of America: Premise.com, https://www.premise.com/how-mask-wearing-has-changed-in-america-a-visual-journey-of-increasing-mask-use/) (2020).

[16] Institute for Health Metrics and Evaluation (IHME), COVID-19: What's new for June 25, 2020 (Seattle, United States of America: Institute for Health Metrics and Evaluation (IHME), University of Washington, https://www.healthdata.org/sites/default/files/files/Projects/COVID/Estimation_update_062520.pdf) (2020).

[17] M Slaoui, Interview with Mary Louise Kelly: Operation Warp Speed top adviser on the status of a coronavirus vaccine (National Public Radio, https://www.npr.org/2020/09/03/909312697/operation-warp-speed-top-adviser-on-the-status-of-a-coronavirus-vaccine) (2020).

[18] K Prem, AR Cook, M Jit, Projecting social contact matrices in 152 countries using contact surveys and demographic data. *PLoS Computational Biology* **13**, e1005697 (2017).

[19] R Patel, IM Longini Jr, ME Halloran, Finding optimal vaccination strategies for pandemic influenza using genetic algorithms. *Journal of Theoretical Biology* **234**, 201–212 (2005).
